# The dental plate on bichir pectoral fins: A unique dermal skeletal element bearing individual odontodes with tooth‐like replacement

**DOI:** 10.1111/joa.70053

**Published:** 2025-09-29

**Authors:** Tomáš Suchánek, Ann Huysseune, Robert Cerny

**Affiliations:** ^1^ Department of Zoology, Faculty of Science Charles University Prague Czechia; ^2^ Research Group Evolutionary Developmental Biology, Biology Department Ghent University Ghent Belgium

**Keywords:** bichir, dental plate, dermal skeleton, odontocomplex, odontodes, scales, teeth

## Abstract

The dermal skeleton appeared early in vertebrate evolution in the form of mineralized skin denticles composed of tooth‐like units—odontodes. This surface odontogenic competence later also expanded inside the oropharyngeal cavity where teeth are formed as modified odontodes possessing innovative replacement dynamics. Whereas in modern cartilaginous fishes, both the internal odontodes (teeth) and external odontodes (scales) exhibit generally the same shape and structure, the dermal skeleton of bony fishes was further modified by the fusion of odontodes forming so‐called odontocomplexes. This ancient dermal armour was reduced in both ray‐finned and lobe‐finned fishes, or disappeared in tetrapods. Bichirs (Polypteridae) occupy a key phylogenetic position as the earliest extant ray‐finned fishes retaining a massive dermal skeleton. We performed developmental and structural analyses of their odontocomplex elements comprising the cranial dermal bones, trunk scales, fin rays, and spines of the dorsal finlets, primarily using the Senegal bichir (*Polypterus senegalus*). All these elements are covered by a hypermineralised layer ganoine, considered to be a true enamel. Yet, during the development of these odontocomplex elements, individual odontodes could not be recognised. However, we also identified one unique dermal element with a dual structural nature combining the scale‐like odontocomplex with individual odontodes. These so‐called dental plates form a narrow series of repeating elements that extend in between the fin rays on bichir pectoral fins. Individual odontodes on these dental plates are organised into C‐shaped rows attached to a scale‐like element. Interestingly, these individual odontodes bear striking morphological and histological similarities to teeth, and their dynamics of replacement parallel that of teeth in bichir oral dentition. Dental plates occupy a distinct dermal skeletal domain on distal pectoral fins, where replacing odontodes form a spiky surface with apparent functional advantages when bichirs rest their pectoral fins upon the substrate.

## Graphical abstract

Pectoral fins of bichirs encompass the so‐called dental plates – unique dermal skeletal elements with individual odontodes identical to the oral teeth.

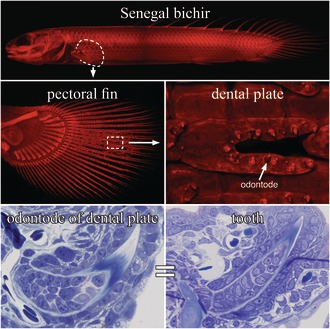

## 1 INTRODUCTION

The early evolution of vertebrates was greatly facilitated by the appearance of a dermal skeletal armour protecting our predecessors from the predation by giant invertebrates (Romer, 1933). The advent of this first mineralised tissue of vertebrates (Donoghue et al., 2006; Donoghue & Sansom, 2002) was apparently promoted by the early skeletogenic ability of the neural crest stem‐cell population (Green et al., 2015; Stundl et al., 2023) and linked with the sensory function of the ancient skin odontodes (Haridy et al., 2025; Houée & Janvier, 2025). The ancestor of skeletonizing vertebrates has been inferred to possess dermal odontodes and polyodontode scales (Donoghue & Sansom, 2002; Janvier, 1996; Keating & Donoghue, 2016). Odontodes as single dermal elements typically consist of a cone of dentine with a superficial enameloid or enamel, developing from a single mesenchymal papilla at the contact zone with a competent epithelial organ (Chen, 2023; Ørvig, 1977). Originally described as comprising all single hard tissue units of the dermal skeleton outside the oropharynx (Ørvig, 1967, 1977), the term odontode was later expanded to include both external odontodes (skin denticles) and internal odontodes (teeth) (Donoghue & Rücklin, 2016; Reif, 1982; Rücklin et al., 2011). Since the phylogenetic distribution of teeth and skin denticles clearly shows that the first odontodes were present in the dermal skeleton, it was classically expected that teeth evolved from ancient external denticles that invaded the mouth at the dawn of gnathostome evolution (Donoghue & Rücklin, 2016; Hertwig, 1874; Reif, 1982). This ‘outside‐in’ extension of the odontogenic competence from the external dermis inward into the oropharyngeal cavity is more recently specified by the ‘modified outside‐in’ hypothesis on the origin of teeth origin (Chen et al., 2020; Huysseune et al., 2009, 2010, 2022; Huysseune & Witten, 2023).

During the course of evolution, individual odontodes merged into larger and more complex dermal skeletal elements, with several models attempting to explain the mechanisms of this major morphological transformation (Donoghue, 2002). In early cartilaginous fishes (Chondrichthyes), polyodontode scales are widespread (Dearden et al., 2021), whereas a dermal skeleton of modern cartilaginous fishes is composed of individual odontodes (placoid scales). This is likely a derived condition that may nevertheless reflect one of the earliest occurring states of the vertebrate dermal skeleton. In contrast, the dermal skeleton of bony fishes (Osteichthyes) is composed of odontocomplexes, formed by superimposition or accretion of individual odontodes (Huysseune & Sire, 1998; Ørvig, 1977; reviewed by Donoghue, 2002). During the further evolution of bony fishes, much of the dermal skeleton was progressively reduced or lost (Sire et al., 2009; Sire & Huysseune, 2003; Vickaryous & Sire, 2009). In the crown group of ray‐finned fishes (Actinopterygii), particularly in teleosts (Teleostei), the odontocomplex is greatly reduced. As demonstrated in zebrafish, only thin layers of dental tissue remain on the scale surfaces (Aman et al., 2023; Aman & Parichy, 2024; Sire & Akimenko, 2004). Among lobe‐finned fishes (Sarcopterygii), odontocomplex, and associated dental tissues have almost entirely disappeared from dermal skeletal elements located outside the oral cavity, with only a few exceptions (Meunier et al., 2019; Vickaryous & Sire, 2009; Williams et al., 2022). Bichirs (Polypteridae), which represent the earliest diverging group of extant ray‐finned fishes, are still possess an elaborate dermal skeleton composed of odontocomplexes, which makes bichirs a crucial subject for comparative research on the evolution of the dermal skeleton (Sire et al., 1998).

Living bichirs encompass 14 species belonging to two genera, bichir (*Polypterus; P. ansorgii*, *P. bichir*, *P. congicus*, *P. delhezi*, *P. endlicherii*, *P. mokelembembe*, *P. ornatipinnis*, *P. palmas*, *P. polli*, *P. retropinnis*, *P. senegalus*, *P. teugelsi*, *P. weeksii*) and reedfish (*Erpetoichthys; E. calabaricus*) (Moritz & Britz, 2019). Recent bichirs inhabit freshwaters of tropical Africa (Moritz & Britz, 2019; Near & Thacker, 2024), but fossil evidence from South America indicates the Gondwanian distribution of this group (Gayet & Meunier, 1991; Meunier & Gayet, 1996). Whereas the origin of bichirs is set to the Devonian period approximately 390 million years ago, living species radiated in the Neogene period, some 19 million years ago (Giles et al., 2017; Near et al., 2014). Bichirs show a number of plesiomorphic features, including embryonic pre‐oral gut and larval stages with cranial adhesive organs and external gills, or a functional lung (Diedhiou & Bartsch, 2009; Kerr, 1907; Minarik et al., 2017). Importantly, bichirs also retained an extensive postcranial dermal armour composed of ancient ganoid scales possessing an odontocomplex (Goodrich, 1907; Kerr, 1952; Sire, 1989; Sire et al., 1998, 2009; Sire & Huysseune, 2003).

The ganoid scale of bichirs consists of four distinct mineralized layers, organized in a manner that optimises resistance to perforation by predators (Bruet et al., 2008). These layers are (1) basal vascular bone on which lies (2) a plywood‐like structure called elasmodine (Sire, 1989), probably derived from the bone of attachment of individual odontodes (Sire, 1990). On top of the elasmodine lies the odontocomplex composed of (3) dentine, organised in one layer covered with (4) ganoine (Sire & Huysseune, 2003). The dentine layer (resembling vasodentine and osteodentine) contains denteons with osteon‐like morphology, whose cavities are tentatively homologous to the pulp cavities of odontodes (Sire & Huysseune, 2003). The ganoine is generally considered to be a multilayered true enamel, homologous to true enamel of lobe‐finned fishes (Kawasaki et al., 2021; Qu et al., 2015; Sire, 1990; Sire et al., 1987). The ganoine is characterised by the presence of small tubercles on its surface, proposed to arise as a result of differences in the secretion process of the epidermis (Coelho et al., 2024; Daget et al., 2001; Gayet & Meunier, 2001; Schultze, 2016). Other dermal skeletal elements of bichirs, such as cranial dermal bones, fin rays, or dorsal spines also carry dental tissues on their surface (Meinke, 1982; Meunier, 1980; Ørvig, 1978; Zylberberg & Meunier, 2013). Importantly, similarly to dermal denticles in skates (Gillis et al., 2017), bichir ganoid scales derive from neural crest cells (Stundl et al., 2023), which typically serve as an embryonic source for oral and pharyngeal teeth (Chen et al., 2020; Fraser et al., 2010). This arguably ancient skeletogenic ability of neural crest cells in the postcranial dermal skeleton is, in typical modern vertebrates, progressively restricted to the cranial region, and bichirs thus might serve as a unique model to study the ancient dermal skeleton of vertebrates.

Here we analyse odontocomplex elements of the bichir dermal skeleton comprising the cranial dermal bones, trunk scales, fin rays, and spines of the dorsal finlets, highlighting evidence for the presence of dental tissues. We specifically focus on a remarkable and often neglected dermal element found only on the pectoral fins in bichirs, the so‐called dental plate (Jarvik, 1959; Sewertzoff, 1932). These dental plates are located on the ventral (inner) side of the pectoral fins, forming longitudinal rows between the fin rays, and a single row at the boundary between the endoskeletal and dermal skeletal domains (Géraudie, 1988). Dental plates have been suggested to represent modified scales (Jarvik, 1959) and were described as being ornamented with vascularised odontodes protruding through the epidermis (Kerr, 1952; Sewertzoff, 1932). Since the dermal skeleton of bichirs is usually formed by odontocomplex elements, the presence of individual odontodes is very intriguing and allows a unique opportunity for comparison with single elements of the internal dermal skeleton–teeth.

## 2 MATERIALS AND METHODS

### 2.1 Rearing and animal collection

All fish were maintained according to institutional and international guidelines for the protection of animal welfare (Directive 2010/63/EU) in our breeding facility at the Department of Zoology, Faculty of Science, Charles University in Prague, Czech Republic. The material analysed encompassed five adults and 15 juveniles of the Senegal bichir (*Polypterus senegalus*), two adults of the Nile bichir (*Polypterus bichir*), one adult of the saddled bichir (*Polypterus endlicheri*), one adult of the ornate bichir (*Polypterus ornatipinnis*), and one sub‐adult of the barred bichir (*Polypterus delhezi*). Adults were obtained from our facility, and juveniles were imported from Aquarium Glaser (Germany) and reared to the desired sizes in our facility. Individuals were sacrificed by an overdose of MS‐222 (Sigma‐Aldrich) and fixed in 4% paraformaldehyde (PFA) for staining of skeletal tissues and for SEM, or in PFA/glutaraldehyde (PG) fixative for histology and TEM. Body size of individuals was measured on fixed material from the tip of the snout to the end of the caudal fin (Total length = TL).

### 2.2 Staining and clearing of skeletal tissues

To analyse the external morphology of the mineralised skeletal elements, specimens were stained with Alizarin Red S (Thermo Scientific Chemicals) and cleared according to Rizzato et al. (2020). Observations were made on a Zeiss Lumar V12 fluorescence stereomicroscope, and photographs were taken by an AxioCam HRm digital camera. For microphotography, the dental plates and scales were removed from stained and cleared specimens and mounted on glass slides in glycerol. Images were captured with an Olympus DP74 camera mounted on an Olympus BX51 compound microscope under epifluorescence illumination, using cellSens 3.1 software.

### 2.3 Histology and electron microscopy

To analyse the internal structure, dermal elements were first demineralised in PG fixative containing 0.1 M EDTA (Merck) for 2 weeks at 8°C, with the solution being changed several times during the process. The samples were further processed for embedding in Epon according to Huysseune et al. (2021). Sections of 2 μm thickness were prepared on a Microm HM 350 equipped with a diamond knife, stained with toluidine blue, and observed on a Zeiss Axio Imager Z1 compound microscope. Images were taken with a Zeiss Axiocam 503 camera and processed using ZEN software. Images in polarised light were captured by an AmScope MF603C‐CCD 6 Mp camera mounted on a Leitz Dialux 22EB microscope.

For transmission electron microscopy (TEM) ultrathin sections of 70 nm thickness were prepared on a Reichert‐Jung UltracutE ultramicrotome, collected on copper grids, contrasted with uranyl acetate and lead citrate solutions, and examined under a Jeol JEM 1010 transmission electron microscope operating at 60 kV. Microphotographs were taken with a Veleta camera.

Specimens for scanning electron microscopy (SEM) were bleached under a light source in 2% potassium hydroxide and 3% hydrogen peroxide in equal proportions, washed, and further digested in 1% trypsin in 2% borax solution for 2–3 weeks to remove the covering epidermis. Next, specimens were dried in an ascending ethanol series, acetone, and liquid carbon dioxide, and coated with gold. Samples were analysed on a JEOL‐JSM‐6380 LV scanning electron microscope operating at 15 kV.

### 2.4 Vital double‐staining of bichir juveniles

Vital double‐staining using Alizarin Red S (Thermo Scientific Chemicals) and Calcein Green (Merck) was performed on Senegal bichir juveniles of 80 mm (*n* = 3) and 120 mm (*n* = 3) TL, according to a modified protocol based on Carnovali et al. (2021). First, the fish were stained by soaking them overnight in a 0.01% Alizarin Red S aqueous solution, which stains the mineralisation front red. The fish were next transferred into tank water overnight to remove excess staining, and again into tank water where they were kept and fed. After 1 or 2 weeks, depending on the desired length between staining experiments, fish were stained overnight with a 0.01% Calcein Green aqueous solution, which labels newly mineralising tissues green. Once again, fish were transferred into tank water overnight to remove excess staining. The fish were kept at 28°C in a darkened tank throughout the experiment. Twenty‐four hours after Calcein Green staining, the fish were anaesthetised by an overdose of MS‐222 (Sigma‐Aldrich) and fixed in 4% PFA overnight at 4°C. The specimens were then washed and digested by 1% trypsin in 2% borax solution for approximately 2 weeks to remove the covering epidermis. Finally, they were transferred into glycerol through an ascending glycerol series. Whole mount fluorescence photographs were taken on a Zeiss Lumar V12 stereo microscope, and final images were stacked using AxioVision 4.0 software. Individual dental plates were separated from the fin and were photographed with an Olympus DP74 camera mounted on an Olympus BX51 compound microscope under epifluorescence illumination, using cellSens 3.1 software.

## 3 RESULTS

### 3.1 Elements of the bichir dermal skeleton

**FIGURE 1:**
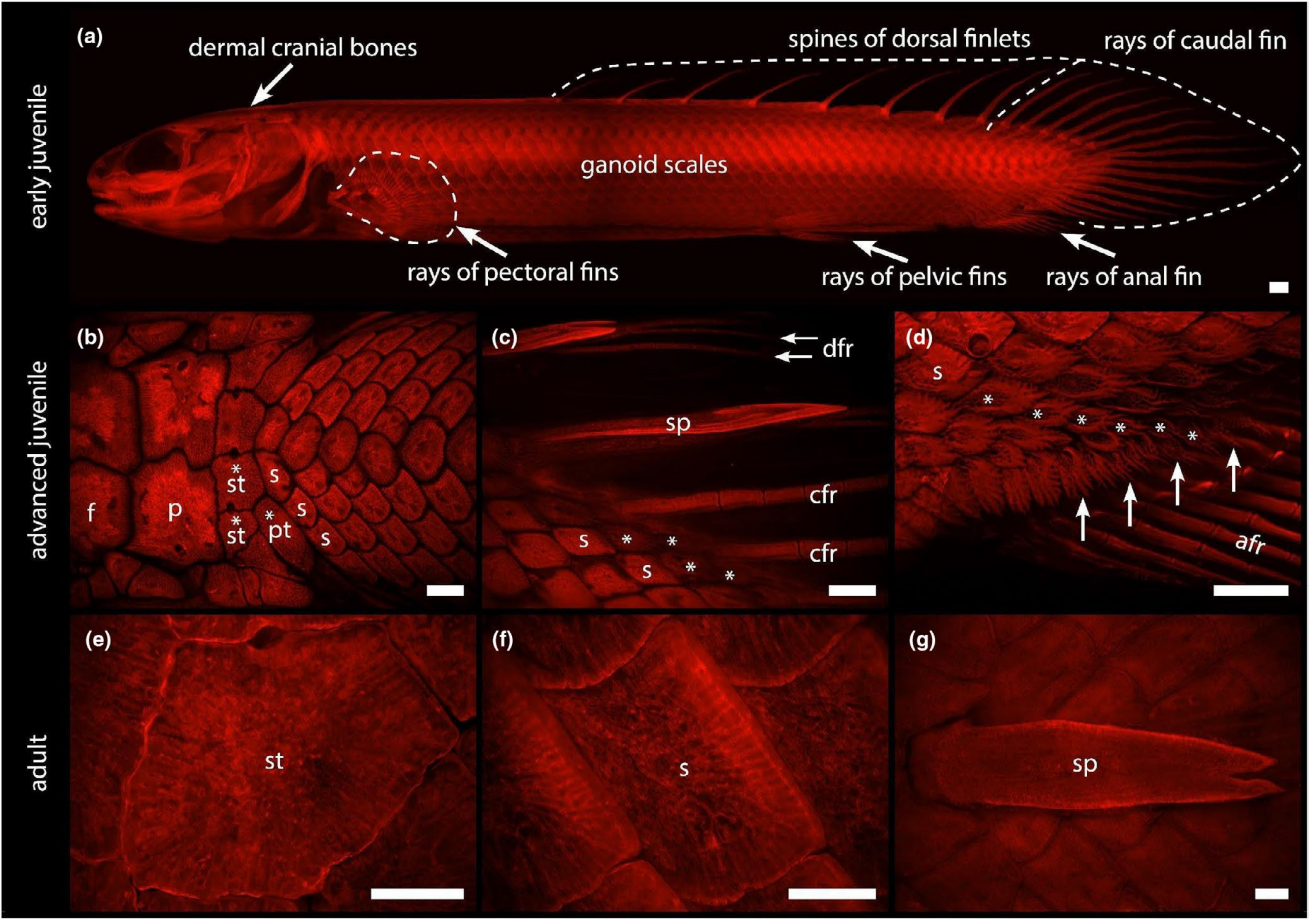
Overview of odontocomplex elements in the Senegal bichir (*Polypterus senegalus*); stained with alizarin and viewed under epifluorescence. (a) General arrangement of odontocomplex elements in an early juvenile (50 mm). The head region is covered by dermal cranial bones, while the trunk is protected by ganoid scales. Spines of the dorsal finlets and fin rays extend outwards from the body. (b) Dorsal view of the head/trunk boundary. Note the posteriormost dermal cranial bones (‘st’, ‘pt’) forming transitional elements between dermal cranial bones (‘f’, ‘p’) and scales of the trunk (‘s’). Head to the left. (c) Lateral view of the interface between spines of the dorsal finlets and rays of the caudal fin. (d) Lateral view of the anal fin showing transitional scales (arrows) with extensions morphologically similar to fin rays. (e) Comparison of a mature cranial bone, one of the supratemporals, and (f) a ganoid scale. (g) Dorsal view of the mature spine of a dorsal finlet. *, scale of transitional shape; afr, fin rays of anal fin; cfr, fin rays of caudal fin; dfr, fin rays of dorsal finlets; f, frontal bone; p, parietal bone; pt., posttemporal bone; s, scale; sp., spine of dorsal finlet; st, supratemporal bone. Scale bars = 1 mm.

The dermal skeleton of bichirs consists of several morphologically distinct elements that encase their entire body, as visualised on a fluorescent image of a whole mount Alizarin Red S stained early juvenile Senegal bichir (*Polypterus senegalus*) (Figure 1). This dermal skeletal armour is mostly composed of flat elements: the dermal cranial bones in the head region, and ganoid scales over the trunk (Figure 1a,b). In contrast, dermal skeletal elements that are extending outwards from the body generally have a simple rod‐like shape. These comprise the fin rays of all paired and unpaired fins, and the spines of the dorsal finlets (Figure 1a,c,d). Unlike in other actinopterygian fishes, the dorsal fin of bichirs is composed of a series of separate finlets that transition posteriorly into the caudal fin rays, within a continuous finfold‐like structure (Figure 1a).

Dermal cranial bones are flat skeletal elements that form the bulk of the skull (Figure 1a). Unlike the overlapping scales of the trunk, dermal cranial bones do not overlap. Each bone is individually named, though the terminology and homology of these bones have long been subjects of debate (e.g., Jollie, 1984). Interestingly, dermal cranial bones at the head–trunk interface tend to be intermediate in shape, size, and pattern of orientation in between cranial bones and trunk scales (Figure 1b). The resemblance between these two main dermal skeletal elements is more obvious at the mature stage when these dermal elements become similarly covered by a ganoine layer (Figure 1e,f).

**FIGURE 2:**
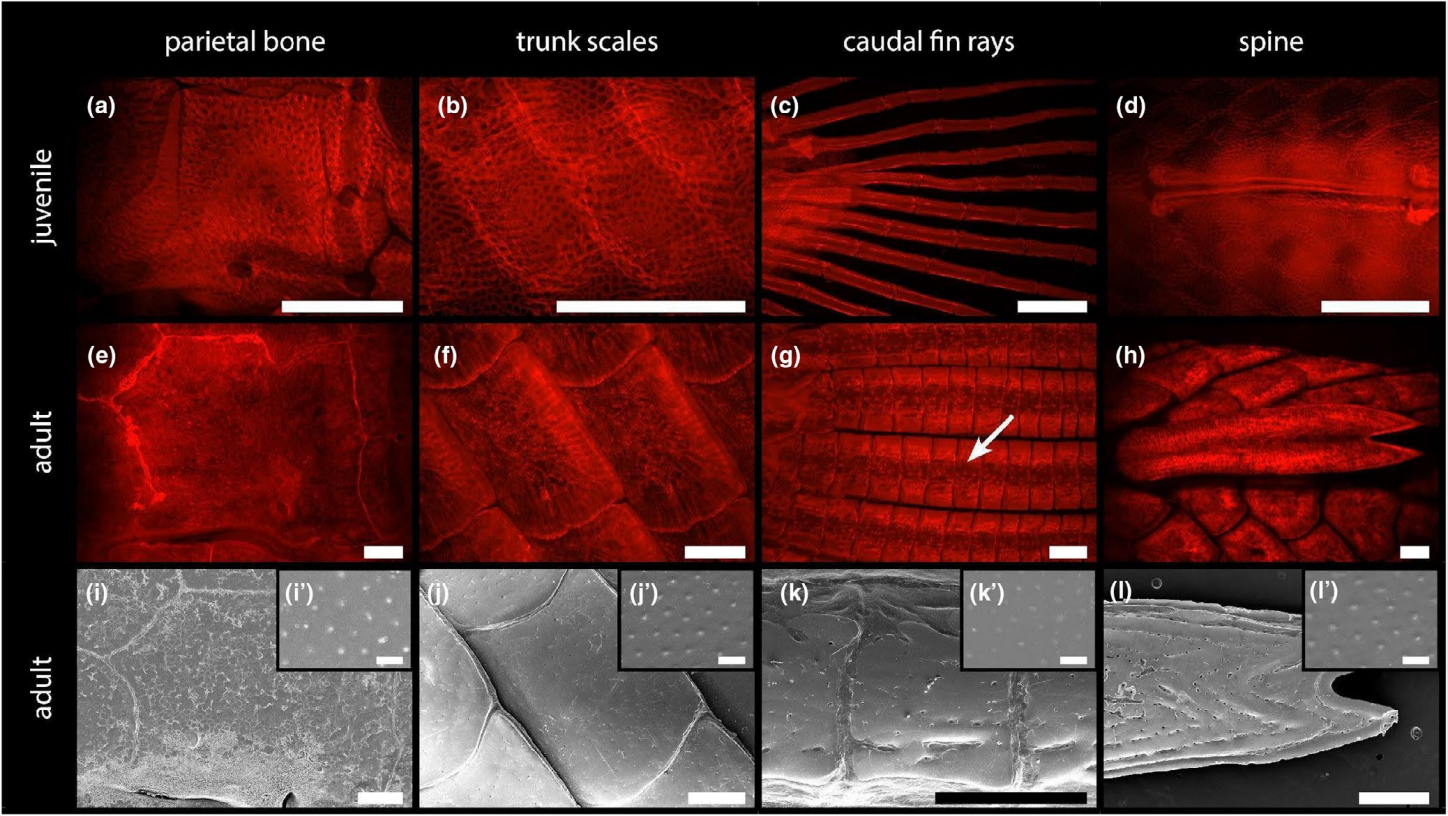
Development of odontocomplex elements in the Senegal bichir (*Polypterus senegalus*). Anterior to the left. (a–h) Stained with alizarin and viewed under epifluorescence. In an early juvenile (50 mm TL), flat odontocomplex elements, that is, dermal cranial bones (a) and ganoid scales (b), start their development as a mineralised meshwork. Elements extending outwards from the body, fin rays (c) and spines (d), start as mineralised rods or double rod‐like structures. Mature odontocomplex elements (e–h) are ultimately covered by hypermineralised ganoine, visible as a glassy tissue on the surface. (i–l) Scanning electron microscope images showing the surface of odontocomplex elements, with details (i’–l’) revealing the presence of tubercles distinctive for ganoine. Scale bars: (a–l) 1 mm; (i’–l’) 10 μm.

The trunk region is completely covered by thick, ganoid scales with a rhomboid shape (Figure 1a,f). They are interlocking, partially overlap, and are arranged in numerous diagonal series, which makes the dermal armour a rigid structure. Interestingly, trunk scales that are situated alongside the finfold region do not display the typical rhomboid shape. Instead, they manifest a rather immature morphology (Figure 1c,d), well comparable to an early stage of a scale (Figure 2b). These distally placed scales exhibit a more elongated shape with longitudinal rods, extending from the scale in an orientation reminiscent of fin rays (Figure 1c,d). This continuous transition in form between scale and fin ray is most prominent near the anal fin where the orientation of the scale mineralised rods parallels the orientation of the fin lepidotrichia (Figure 1d, arrows). The spines of the dorsal finlets form a single, solid, undivided element (Figure 1g), in contrast to the fin rays, which are segmented. This also allows for the distinction between the spines and fin rays of the caudal fin in the dorsocaudal region of the bichir (Figure 1a).

### 3.2 Development of dermal odontocomplex elements

We next decided to analyse early stages of formation of the bichir elements to assess the possible presence of individual odontode‐like structural units therein. Both types of flat elements, that is, dermal cranial bones (Figure 2a) and ganoid scales (Figure 2b), start their formation as a mineralised meshwork which foreshadows the final shape of these elements. On the other hand, elements that extend out of the body, that is, fin rays (Figure 2c) and spines (Figure 2d), are initially composed of individual mineralised rods only.

In adult bichirs, all these elements are covered by a tissue of glassy nature – a hypermineralised ganoine layer (Figure 2e–h). The glassy character is apparently due to the lack of staining with Alizarin Red, a characteristic observed before for hypermineralised tissues such as enamel or enameloid (Bruneel et al., 2015). We further investigated the surface of mature elements using SEM (Figure 2i–l). Higher magnifications revealed the presence of tiny tubercles on the surface (Figure 2i’–l’) confirming the ganoine, pointing to a shared, deep origin of these elements from individual odontodes. All analysed elements of the dermal skeleton contain ganoine, indicating the presence of an odontocomplex, and henceforth we refer to them as odontocomplex elements.

### 3.3 The dental plate: A unique dermal element with a dual nature

**FIGURE 3:**
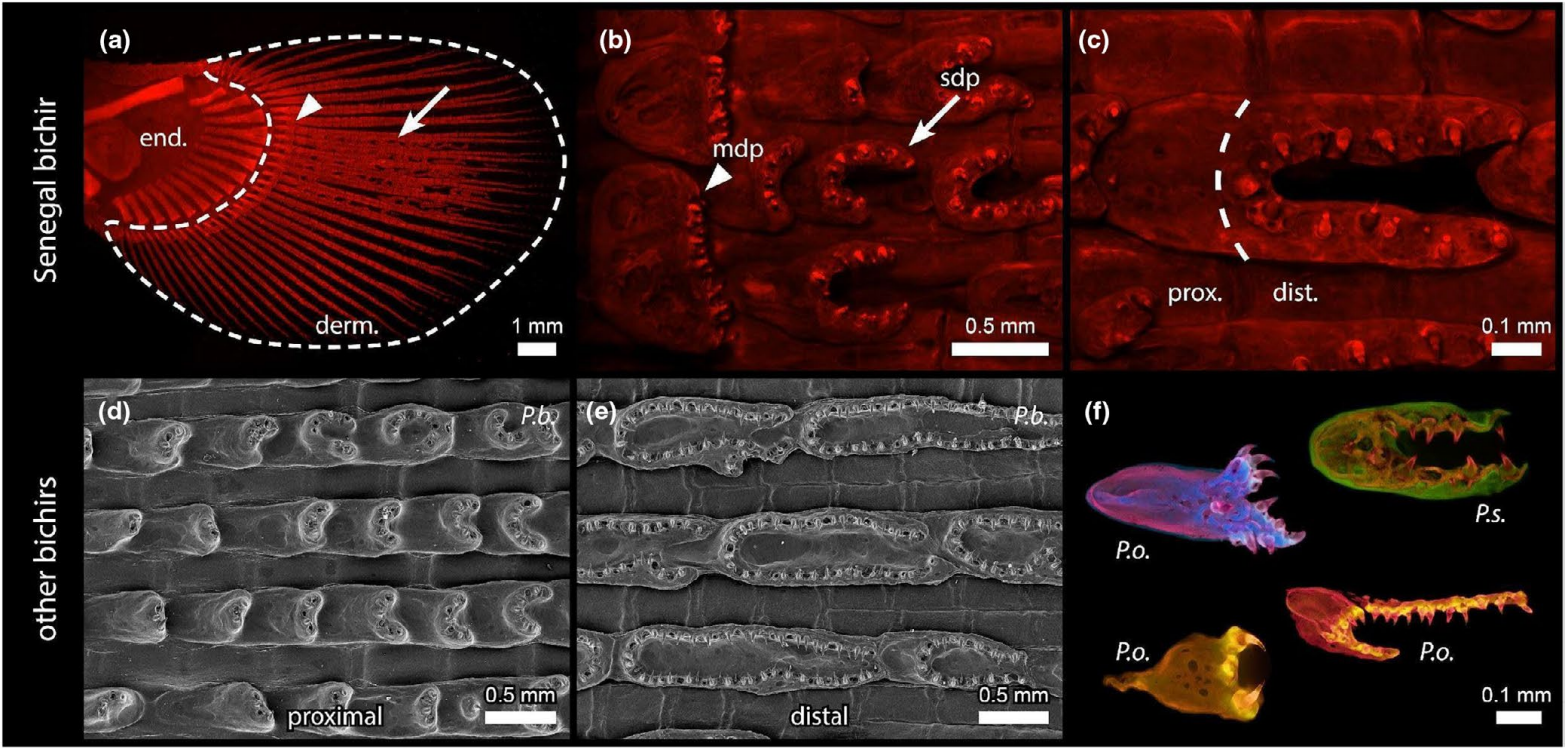
Dental plates on the pectoral fins of the Senegal bichir (*Polypterus senegalus*) unless otherwise stated. (a–c, f) Stained with alizarin and viewed under epifluorescence. (a) Dermal skeleton domain of the pectoral fin (dashed line) housing the marginal dental plates (arrowhead) at the border and serial dental plates (arrow) between fin rays. (b) Detail of the border between endo‐ and dermoskeletal domains of the fin with marginal and serial dental plates. (c) Detail of the serial dental plate consisting of a proximal part (body) and a distal odontode‐bearing part. (d, e) Scanning electron microscope images showing the shape of the serial dental plates varying according to their proximodistal location along the fin (*Polypterus bichir*). (f) Serial dental plates exhibit a tremendous variation in shape within one, as well as across species (*P. ornatipinnis*, *P. senegalus*). Derm, dermal skeleton domain; dist, distal part of the dental plate; end, endoskeletal domain; mdp, marginal dental plate; P. o., *Polypterus ornatipinnis*; P. s., *Polypterus senegalus*; prox., proximal part of the dental plate; sdp, serial dental plate. Scale bars as indicated.

Our structural analysis identified one dermal element, the dental plate, that exceptionally combines a scale‐like odontocomplex with individual tooth‐like odontodes (Figure 3). These dental plates are located solely on the pectoral fins, in between lepidotrichia of the distal dermal skeletal domain (Figure 3a, dotted line). In the Senegal bichir, the dental plates appear to be restricted to the central area of the fin (Figure 3a, Figure S1). However, in other adult bichir species, dental plates are present throughout the entire dermal fin domain (Figure S1). Intraspecific differences in the Senegal bichir were not observed in the distribution of dental plates, which were consistently restricted to the central part of the fin, yet the morphology of the individual dental plates was highly variable. Interestingly, aside from *Polypterus delhezi*, in which the dental plates are present on both sides of the fin (Figure S2a), all other bichir species analysed possess dental plates only on the ventral side of their pectoral fins (Figure 3, Figure S1).

Dental plates generally occur as two types: the marginal (or transversal) dental plates are located at the border between endoskeletal and dermal skeletal domains of the fin, forming a single line of elements that borders the endoskeletal domain of the fin (Figure 3a,b, arrowhead). In contrast, the serial (or longitudinal) dental plates are present in long rows that alternate with fin rays along the entire fin (Figure 3a,b, arrow). In the Senegal bichir, the marginal dental plates start to appear in juveniles of 75 mm TL. They first form in the medial part of the pectoral fins and subsequently expand laterally during later ontogeny (Figure S3). However, the serial dental plates start to appear later, in juveniles of 85 mm TL, and gradually fill the medial part of the fin in a proximodistal direction (Figure S3).

Regardless of the type, all dental plates generally consist of a flat proximal body and a distal part with individual odontodes (Figure 3c). In the case of the marginal dental plates, individual odontodes form a single line alongside the distal side of this element (Figure 3b). In the case of the serial dental plates, individual odontodes tend to form long C‐shaped series, yet show a great variation along the proximodistal axis of the fin (Figure 3d,e, Figure S1). Moreover, individual dental plates can merge, especially in the distalmost part of the fin, leading to the formation of varied shapes deviating from the typical C‐shape (Figure S2b). Serial dental plates also reveal a surprising morphological variation across different bichir species (Figure 3f, Figure S1).

### 3.4 The body of the dental plate has a scale‐like shape and structure

**FIGURE 4:**
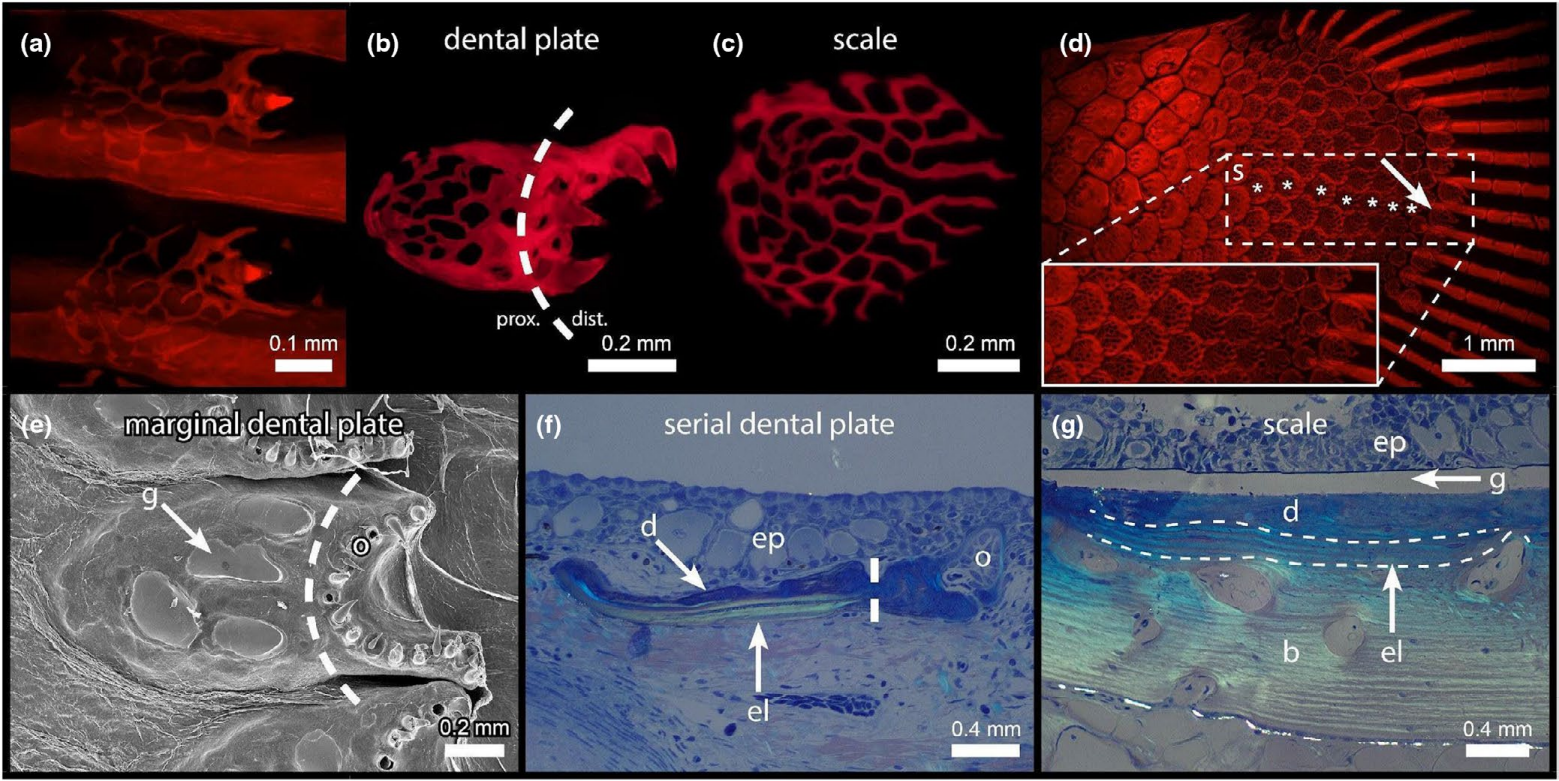
The body of the dental plate has the structure of a scale. The Senegal bichir (*Polypterus senegalus*), unless otherwise stated. (a–d) Stained with alizarin and viewed under epifluorescence; (f, g) semithin sections viewed under polarised light. (a) Earliest developmental stage of the dental plate, composed of a flat body and one or a few attached odontodes. (b) Early developmental stage of the body of the dental plate displays a mineralised meshwork similar to that observed in the early developmental stage of the scale (c). (d) A series of immature scales (asterisks) and a transitional scale (arrow) on the dorsal side of the pectoral fin in a juvenile (enlarged in the box), illustrating the transition of scales extending to dental plates between the fin rays on the ventral side. (e) Scanning electron micrograph of the mature marginal dental plate of *P. endlicheri* with patches of ganoine. (f) Body of a marginal dental plate contains an elasmodine layer, distinguishable in polarised light. (g) Internal structure of a mature ganoid scale showing a layer of elasmodine visible in polarised light together with the lamellar bone below. *, transitional scale; b, bone; d, dentine; el, elasmodine; ep, epidermis; g, ganoine; o, odontode; s, scale. A dashed line indicates the boundary between the proximal (prox.) and distal (dist.) part of the dental plate. Scale bars as indicated.

The dental plate is unique among all other bichir dermal skeletal elements in its apparent dual structural nature. From the earliest mineralised stage onwards, the dental plate consists of a proximal flat body, with a single or a few individual odontodes attached distally (Figure 4a). The early body of the dental plate is composed of a mineralised meshwork (Figure 4b) bearing striking morphological similarities to the early mineralised stage of a bichir ganoid scale (Figure 4c). Interestingly, in bichir juveniles, the scales of the endoskeletal domain of the pectoral fins (present only on the dorsal side, with the exception of a small area on the ventral side; Figure S4c) reveal a series with seemingly decreasing maturity as they are located closer to the edge of the endoskeletal domain (Figure 4d). Immature scales that are situated at the edge of the endoskeletal domain of the pectoral fin (Figure 4d arrow) then clearly resemble the early body of the (marginal) dental plates on the corresponding ventral side.

In the case of the marginal dental plates, the proximal scale‐like part is later covered with ganoine (Figure 4e), like on other odontocomplex elements (Figure 2). However, we never detected a ganoine layer on any of the serial dental plates in any bichir species we analysed. Moreover, in the case of the marginal dental plates, we also detected an elasmodine layer in the internal structure of their bodies (Figure 4f), which is otherwise known only from ganoid scales (Figure 4g).

### 3.5 Odontodes of the dental plate have a tooth‐like form and structure

**FIGURE 5:**
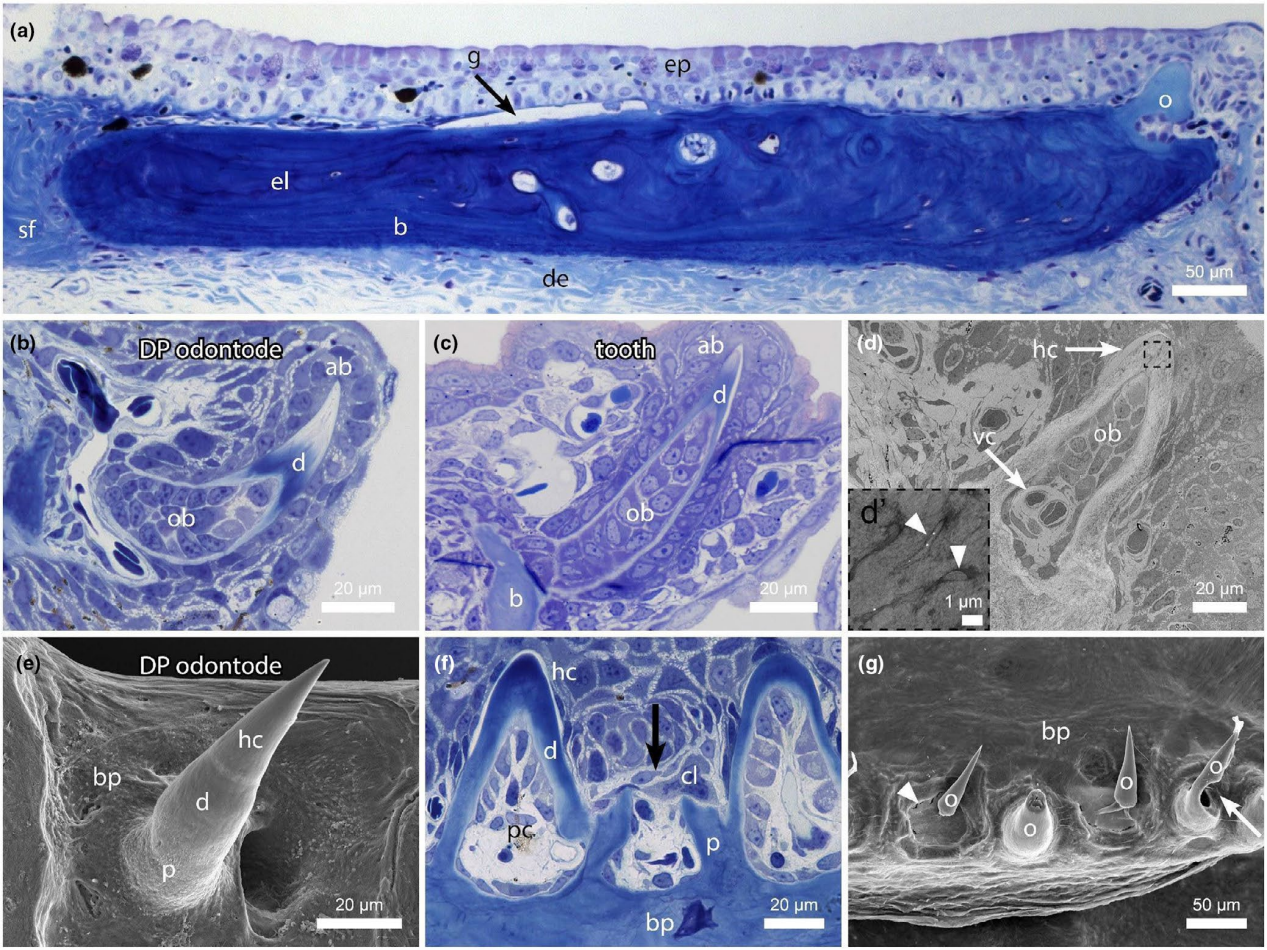
Individual odontodes of the dental plate in the Senegal bichir (*Polypterus senegalus*) have the structure of a tooth. (a, b, c, f) Semithin sections viewed under transmitted light; (e, g) scanning electron micrographs. (a) Section through a mature marginal dental plate, revealing its internal tissue organisation, with an odontode located at its distal edge. Note Sharpey's fibres firmly anchoring the marginal (but not serial) dental plate in the dermis. (b) A developing odontode of a serial dental plate is formed by cells that are anatomically identical to those in developing mandibular teeth of an embryo (stage 34), as shown in (c). (d) TEM micrograph of a dental plate odontode showing a vascular canal (arrow) entering its pulp. Dentinal tubules (arrowheads) are visible under higher magnification in the indicated area. (e) Odontodes of the dental plates are composed of morphologically distinct tissues similar to those in teeth. (f) Odontodes of the dental plates are resorbed (arrow) by multinucleated clast cells. (g) Odontode resorption (arrow) follows a typical pattern, also observed in teeth. Replacement odontodes form in the same location as their predecessors (arrowhead). Ab, ameloblasts; b, bone; bp, basal plate; cl, clastic cell; d, dentine; de, dermis; el, elasmodine; ep, epidermis; g, ganoine; hc, hypermineralised cap; o, odontode; ob, odontoblasts; p, pedicel; pc, pulp cavity; sf, Sharpey's fibres; vc, vascular canal. Scale bars as indicated.

Across all dermal skeletal elements, individual odontodes appeared to be present only on dental plates. This called for a more detailed analysis and for a comparison to other individual odontodes, notably teeth (Figure 5). Dental plates are located within the dermis and covered by epidermis (Figure 5a). Odontodes are attached to the distal edge and also partly covered with epidermis. Once fully formed, odontodes protrude through the epidermis (Figure S2c). Histological comparison of a single odontode from a dental plate (Figure 5b) with a mandibular tooth from the oral dentition (Figure 5c) revealed striking similarities. Like teeth, dental plate odontodes are formed by a dentine cone surrounding a pulp cavity. The dentine is covered by non‐staining, hypermineralised tissue. Both odontode and tooth are surrounded by an epithelium, with distinctively differentiated ameloblasts surrounding the tooth tip. Dentine‐forming odontoblasts are located in a vascularised pulp (Figure 5b,c). TEM of a single odontode of a dental plate clearly showed the presence of a hypermineralised cap, as well as a well‐developed vascularization underlying the pulp cavity (Figure 5d). At higher magnification, the presence of tubules within the dentine is visible (Figure 5d’, arrowheads). Scanning electron micrographs of a dental plate odontode clearly revealed a border between the apical hypermineralised tissue and the dentine cone. Moreover, they showed the distinctive presence of another tissue by which the odontode is attached to the underlying basal plate (Figure 5e). This cylindrical structure, or pedicel, forms in the prolongation of the dentine base. The basal plate becomes incorporated into the matrix of the dental plate and can be recognised by its different staining properties in histology (Figure 5a).

Like teeth, odontodes of the dental plate undergo resorption. This process starts at the base of the odontode and is performed by clast cells (Figure 5f). It seems that the odontode tip is not shed but rather resorbed within the epidermis (Figure S2d), similar to what has been described for teeth in the oral dentition of Atlantic salmon (*Salmo salar*) (Huysseune et al., 2007). Whole mount SEM views revealed prominent resorption activity along the series of odontodes, with new odontodes forming in place of previous ones (Figure 5g, arrowhead). Incompletely removed pedicels may persist and become overgrown by new pedicels.

### 3.6 Odontodes of the dental plate have a high turnover mirroring the dynamics of the bichir dentition

**FIGURE 6:**
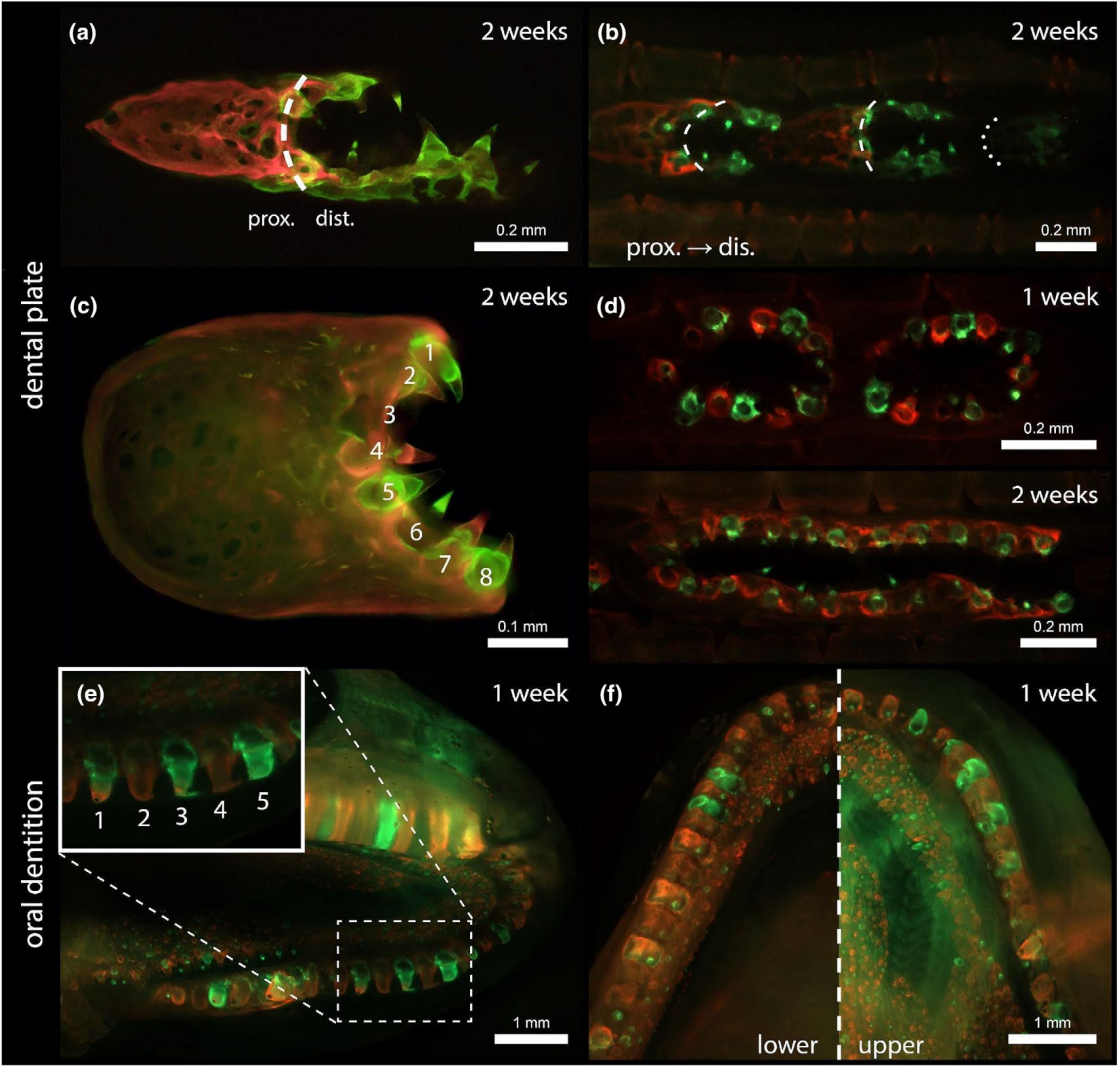
Odontodes of the dental plate in the Senegal bichir (*Polypterus senegalus*) have the dynamics of a dentition as revealed by vital staining. Stained with alizarin and calcein and viewed under epifluorescence. (a) Proximal part initially stained red and distal part subsequently stained green shows proximo‐distal growth of a serial dental plate within a 2‐week interval. (b) Proximodistal patterning similar to that shown in (a) across a row of serial dental plates. (c) Detail of a serial dental plate, showing similar stages of formation of odontodes every third position (1 and 4, 2 and 5). (d) Double‐stained serial dental plates show new and ongoing formation of odontodes in nearly all positions after a 2‐week interval between both staining events, compared with only in alternating positions after a 1 week interval. (e) Teeth of the maxilla show ongoing odontode formation in alternating positions. (f) Oral teeth are replaced in alternating positions (with a shift in the cycle between upper and lower jaw), thus resembling the turnover observed in dental plate odontodes (compare with d, upper panel). dist., distal part of the dental plate (in a) or of the pectoral fin (in b); Prox., proximal part of the dental plate (in a) or of the pectoral fin (in b). Scale bars as indicated.

Individual odontodes of the dental plates appear structurally identical to the oral teeth of bichirs, which led us to also compare the dynamics of appearance and loss or replacement of these skeletal elements. We used vital double‐staining with Alizarin Red followed by Calcein Green after 1 or 2 weeks for monitoring mineralised tissue deposition in Senegal bichir juveniles (Figure 6). Using this protocol, a first‐formed matrix is labelled red, while a matrix that is deposited 1 or 2 weeks later is labelled green.

After double‐staining, early dental plates showed a body entirely labelled red, while odontodes attached to its distal edge showed green label only (Figure 6a). This indicates that the body forms first, followed by the formation of the odontode‐bearing distal part. This apparent proximodistal sequence was also observed repeatedly along a series of forming serial dental plates (Figure 6b). Labelling with two different stains can furthermore reveal different aspects of individual odontode formation. Odontodes stained green have been mineralising in the last day and are therefore recently formed, while odontodes stained red were mineralising 1 or 2 weeks earlier and are therefore older. The presence of double‐labelled odontodes after a 2‐week interval indicates that odontode formation and their attachment took at least 2 weeks. Double‐labelled odontodes always display green proximal to red, confirming centripetal matrix deposition, just like in teeth (Figure 6c, e.g. n°7).

Using the above information, it is possible to trace the dynamics of odontode addition and replacement. Odontodes at the distal end are always labelled green, indicating addition at the end of the row. Given this sequential addition in a proximodistal direction, green‐labelled odontodes amidst red‐labelled odontodes are the result of replacement. The pattern of replacement, however, varies in the different dental plates examined. Close inspection of early dental plates reveals that odontodes form a series of increasing age across a set of three odontodes (Figure 6c). Thus, in the example shown, odontode 3 is the youngest, followed by 2 and 5, and then by odontodes 1 and 4. In older dental plates, with the two staining events separated by 1 week, odontode positions labelled green alternate with odontodes labelled red (Figure 6d, upper panel). Close inspection reveals that several of the green‐labelled odontodes have a red tip or represent the mineralising pedicel, indicating that approximately half of the odontodes are in a stage of ongoing development. After a 2‐week interval between the two staining events, green‐labelled odontodes or pedicels are present in almost every position (Figure 6d, lower panel). Some entirely red odontodes have persisted, but most red pedicels are now associated with a forming (green) odontode tip. This indicates that odontode formation is ongoing in almost all positions (Figure 6d, lower panel). Together, the results after a 1‐ and a 2‐week interval point to a turnover rate of 2 weeks or a little more, consistent with observations on double labelling. This represents a rather unexpectedly high turnover of dental plate odontodes. Replacement of odontodes likely comes to a halt at some point, as we observed a Senegal bichir ~17 years old and measuring 310 mm in total length, whose marginal dental plates lacked odontodes (Figure S4b).

Because the dental plate odontodes exhibit many features shared with teeth, we assessed the developmental and replacement dynamics of the oral teeth from the same juveniles. In bichirs, nearly, all bones in the oropharyngeal cavity are equipped with homodont, single conical teeth (Clemen et al., 1998). On the outer tooth arcade, where teeth reach the largest size (Figure 6e,f), teeth of similar developmental stages (either red‐ or green‐labelled) alternate (Figure 6e, positions 1, 3, 5), comparable with odontodes in the dental plates (compare with Figure 6d). Thus, within 1 week, approximately half of all teeth are newly formed or their formation is ongoing. Moreover, teeth in the outer arcade of the lower jaw are in a different part of the cycle when compared with their upper jaw counterpart (Figure 6f). Thus, the turnover rate of the dental plate odontodes and of the jaw teeth seems comparable, similarly to their alternating pattern. In other tooth locations, like the inner tooth arcade or palatal bones, the size of the teeth varies relative to their location within the oropharynx, and tooth numbers are accordingly high, rendering it difficult to recognize specific patterns (Figure 6f).

Together, these data reveal that individual odontodes from the pectoral fins of bichirs have a surprisingly high turnover rate that resembles the dynamics of teeth within the oral dentition. Moreover, the alternating pattern of replacement also bears striking similarities between dental plate odontodes and teeth.

## 4 DISCUSSION

In this study, we provide a developmental and morphological analysis of various dermal skeletal odontocomplex elements in bichirs, including dermal cranial bones, trunk scales, fin rays, and spines. Interestingly, we also identified one dermal skeletal element with a dual structural and dynamic nature that combines both the scale‐like odontocomplex and individual tooth‐like odontodes. These dental plates are curiously situated on bichir pectoral fins and are ornamented with a series of replacing odontodes that bear striking morphological, histological, and dynamic similarities to the oral teeth of bichir.

### 4.1 The body of the dental plate is an immature scale

The body, or proximal part of the dental plate, is composed of an odontocomplex. Initially, it forms a mineralised meshwork, which was previously identified in scales as a dentine (Sire, 1989; Sire et al., 2009). The meshwork of the early dental plates is comparable to the meshwork of a typical ganoid scale (Figure 4) in terms of morphology (shape and size) and development. Also, both scales and marginal dental plates contain a layer of highly organised elasmodine, which is missing in other odontocomplex elements (Gayet et al., 1997; Meinke, 1982; Zylberberg & Meunier, 2013). Moreover, similar to ganoid scales, marginal dental plates are ultimately covered by hypermineralised ganoine and attached to the underlying dermis by Sharpey's fibres (Figure 5a). On the contrary, the serial dental plates are missing both elasmodine and ganoine layers, but also an attachment by Sharpey's fibres, and thus seemingly represent immature or underdeveloped scales. Importantly, this structural reduction of the serial dental plates leads to a less rigid structure, allowing more flexibility in the distal dermal skeletal domain of the pectoral fin (Figure 3a, Figure S1).

The close relationship between scales and dental plates is particularly well visible on the ventral side of the pectoral fins (Figure S4c). There, some scales can be observed bearing few odontodes, and as such, they seem to represent transitional elements between scales and dental plates, also by their shape. Another case of transitional scales was observed on the dorsal side of the pectoral fins (Figure 4d). There, a series of mature to less developed scales with the morphology of dental plates forms a clear morphocline. This supports the statement that dental plates could represent derived scales ornamented by individual odontodes (Jarvik, 1959).

Almost 150 years ago, structural similarities between osteichthyan scales and segments of fin rays led some authors to suggest that these elements represent homologous structures (Goodrich, 1904; Hertwig, 1876, 1879). Other authors elaborated on this hypothesis further, stating that all dermal elements might have evolved by differentiation of one ancestral element (Jarvik, 1959; Schaeffer, 1977). More recently, this hypothesis was supported by the fossil evidence from the stem ray‐finned fish *Cheirolepis*, where scales and fin rays show noticeable structural similarity (Zylberberg et al., 2016). This hypothesis is also supported by our data, which reveal a similar structural composition of the odontocomplex elements, as well as a gradient in morphological similarity between scales and other dermal elements at their points of contact. All dermal elements in bichir, with the exception of teeth, have been claimed to possess an odontocomplex in their structure (Sire et al., 1998), which is believed to result from the fusion of individual odontodes. The odontocomplex is composed of two dental tissues: (1) superficial ganoine, distinctive by small tubercles (Figure 2) (Coelho et al., 2024; Daget et al., 2001; Gayet & Meunier, 2001; Schultze, 2016), considered homologous to the enamel of lobe‐finned fishes (Kawasaki et al., 2021; Qu et al., 2015; Sire, 1990; Sire et al., 1987); and (2) dentine, which underlies the ganoine. Our developmental analysis revealed that the early mineralisation stage of the flat odontocomplex elements, that is, dermal cranial bones and scales, shares the mineralised meshwork as a common feature (Figure 2). Moreover, at the contact zones between different odontocomplex elements, a gradient of intermediate shapes was observed, further underscoring the close affinity between different dermal elements. This is especially striking at the head/trunk interface, where the most posterior lines of dermal cranial bones (e.g., supratemporals and posttemporals sensu Rizzato et al., 2020) form clear transitional elements between dermal cranial bones and trunk scales (Figure 1b).

In conclusion, bichir odontocomplex elements share a structural composition of dental tissues, ganoine, and dentine. These elements develop in a similar manner, with flat odontocomplex elements characterised by a mineralised meshwork. Transitional elements present at the interface between different dermal elements further support a common origin of all odontocomplex elements, produced by a shared developmental mechanism rooted deep in the evolution of the dermal skeleton, possibly even derived from a single structure.

### 4.2 Odontodes of the dental plate display tooth‐like replacement

Our analysis of the individual odontodes that are ornamenting the distal part of bichir dental plates revealed surprising structural and developmental similarities with oral teeth (Figure 5). Structurally, odontodes of the dental plates are composed of tubular dentine surrounding a pulp cavity, just like teeth. They are capped with a hypermineralised tissue and attached to the skeletal support by a mineralised pedicel. The hypermineralised cap appears to be formed of enameloid, considering its formation from the tip to the bottom, same as in teeth (Wacker et al., 2001). However, in the dental plate odontodes, we have no evidence for the possible presence of collar ganoine, which has been shown to cover both the tooth shaft and the enameloid cap of bichir teeth (e.g., Kawasaki et al., 2022). Also, in contrast to odontocomplex elements which typically remain covered by epidermis (Huysseune & Sire, 1998), mature odontodes of bichir dental plates penetrate the epidermis, as do teeth, as well as skin odontodes in teleosts, or placoid scales of chondrichthyans (Meyer & Seegers, 2012; Sire et al., 2009).

Interestingly, odontodes of the dental plate exhibit rapid and continuous replacement, comparable with the replacement of oral teeth (Figure 6). Alternate replacement dominates, as in many vertebrate dentitions (Edmund, 1960; Osborn, 1974, reviewed by Huysseune & Witten, 2023), although replacement every third position is also observed. Both patterns are also observed in Atlantic salmon (Huysseune et al., 2007). Continuous replacement is common for teeth (reviewed in Berkovitz & Shellis, 2023; Huysseune & Witten, 2023), but uncommon for extraoral odontodes. In chondrichthyans, extraoral odontodes, called placoid scales, are replaced only after wounding (Martin et al., 2016). In the teleost *Ancistrus*, odontodes, called dermal denticles, possess only two generations (Mori & Nakamura, 2022). Tooth replacement probably originated in stem‐osteichthyans, for example, *Andreolepis* (Chen et al., 2016), whereas stem gnathostomes did not replace their dentition at all (Vaškaninová et al., 2020). Interestingly, expression of the gene *sox2* is typically absent in extraoral odontodes, as shown in the catshark *Scyliorhinus* or catfish *Ancistrus* and is perhaps linked to the absence of replacement of extraoral odontodes (Martin et al., 2016; Mori & Nakamura, 2022). Yet, teeth and extraoral odontodes are classically considered homologous structures (Debiais‐Thibaud et al., 2011). Two exceptions are known in teleosts, where extraoral odontodes continue to be replaced: the armoured catfish *Corydoras aeneus* (Sire & Huysseune, 1996) and the denticle herring *Denticeps clupeoides* (Sire et al., 1998), in which, however, the replacement is not as rapid as in teeth. Moreover, in both these species, after the odontodes are shed, pedicels persist and are embedded in the bony matrix, and new odontodes form on top. The same process is also observed in the dental plates. This repetitive shedding of odontodes followed by the embedding of their pedicels leads to the formation of basal plates with typical inner organisation.

Teeth develop through interactions between neural crest‐derived mesenchyme and epithelium competent for tooth formation, regardless of whether the epithelium originates from ectoderm or endoderm (Fraser et al., 2010; Soukup et al., 2008, 2021). Such conditions were also established in the pectoral fins of the bichir, resulting in the formation of dental plate odontodes. Data presented here clearly show that these odontodes have the same structure as oral teeth, including, surprisingly, rapid and continuous turnover.

### 4.3 Functional advantage of the dental plates in bichirs

The dental plates comprising a series of replacing odontodes are curiously situated only on bichir pectoral fins. Here, they seem to play an important role in the context of their locomotion and in the characteristic position when the animal rests, as famously depicted in the first illustration of a *Polypterus* larva (Budgett, 1901; Hall, 2001). Bichirs commonly use their pectoral fins as a support for the body to move across the substrate in their terrestrial‐like locomotion (Standen et al., 2014, 2016). We suggest that the series of dental plates on the ventral surface of the fin serves as an anti‐slip pad. Moreover, odontodes of the dental plate possess a rapid turnover, which is similar to the oral teeth in the mouth of bichir, permanently securing a series of sharp individual units. It is crucial for teeth to be sharp at all times for prey capture, while in the pectoral fins, sharp odontodes likely maintain a proper anti‐slip function. Interestingly, the pectoral fins of bichir can regenerate (Cuervo et al., 2012; Darnet et al., 2019). At present, it is unknown if the dental plates regenerate as well, and whether that includes their odontode part. Irrespective of whether this is the case, dental plates on the bichir pectoral fins show great promise as an accessible model to study tooth‐like replacement outside the mouth.

## AUTHOR CONTRIBUTIONS

**Tomáš Suchánek:** concept, acquisition of data, data analysis, drafting of the manuscript, approval of the article. **Ann Huysseune:** data analysis, critical revision of the manuscript, drafting of the manuscript, approval of the article. **Robert Cerny:** concept, data analysis, drafting of the manuscript, approval of the article.

## FUNDING INFORMATION

This study was supported by funding from the Charles University Grant Agency (GAUK) (258122) to TS. We also acknowledge a grant from The Czech Science Foundation GACR (project no. 22‐25061S), and are also thankful for the support provided by Charles University grant SVV 260685/2025.

## CONFLICT OF INTEREST STATEMENT

The authors declare no conflicts of interest in association with the present study.

## Supporting information


Figures S1–S4

## Data Availability

The data that support the findings of this study are available on request from the corresponding author. The data are not publicly available due to privacy or ethical restrictions.
