# Supplementary material for: The dental plate on bichir pectoral fins: A unique dermal skeletal element bearing individual odontodes with tooth‐like replacement
Source: J Anat. 2025 Sep 29;249(1):127–40. doi: 10.1111/joa.70053 (PMC13238799; doi:10.1111/joa.70053)
Supplement: Supplementary file 1 — Figures S1–S4 [file JOA-249-127-s001.docx]

Supplementary Figures

**
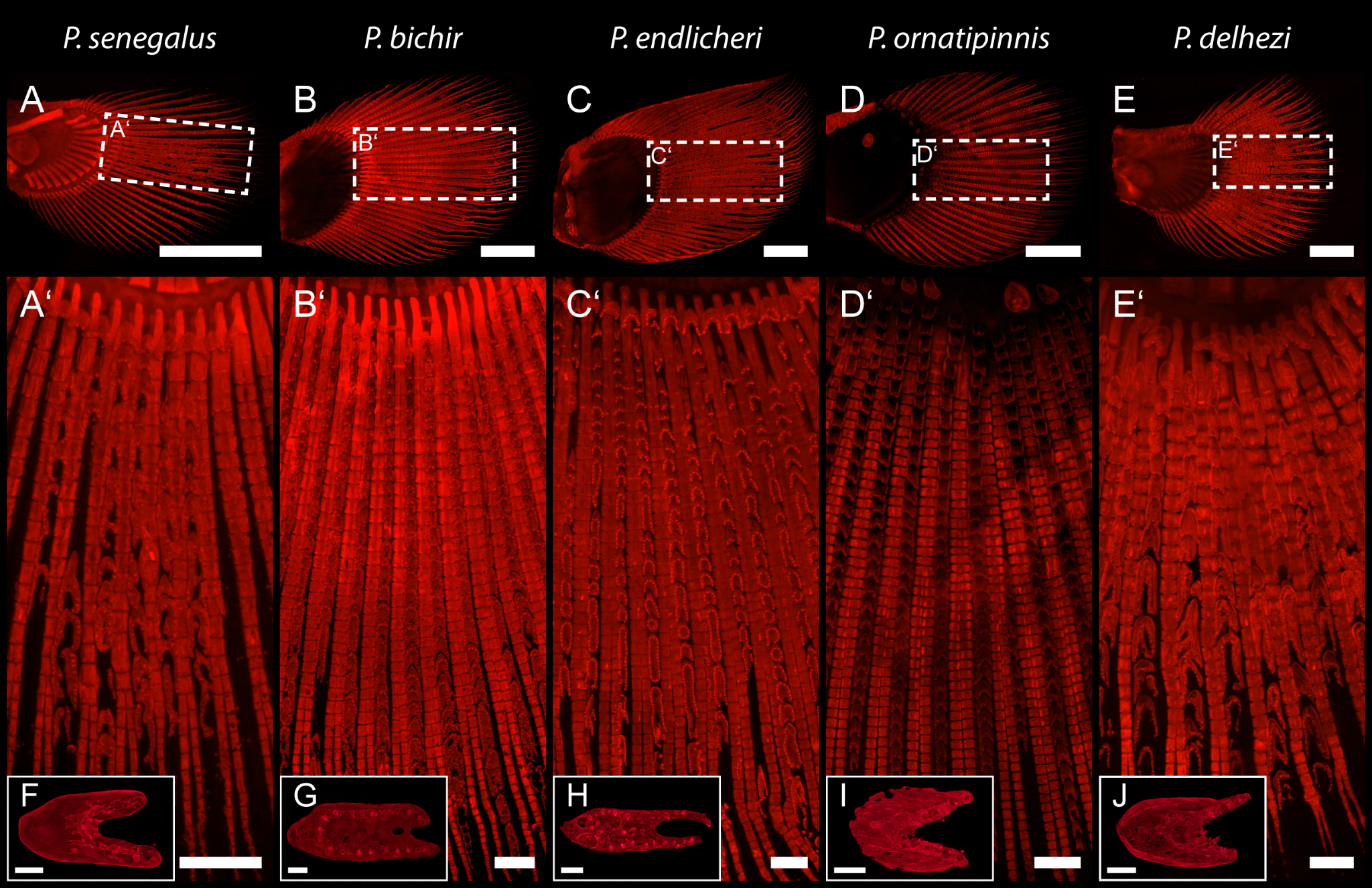
**

**Suppl. fig. 1 Interspecific differences of distribution of dental plates on pectoral fins in polypterids.** Serial dental plates are distributed between fin rays only in the middle part of the fin in Senegal bichir (A), while in other polypterids, such as *P. bichir* (B), *P. endlicheri* (C), *P. ornatipinnis* (D) and *P. delhezi* (E) they are distributed throughout the fin. Details of the boxed areas in (A’-E’). Individual serial dental plates of the corresponding species in (F-J). Scale bars: A-E = 5 mm; A’-E’ = 1 mm; F-J = 200 μm.


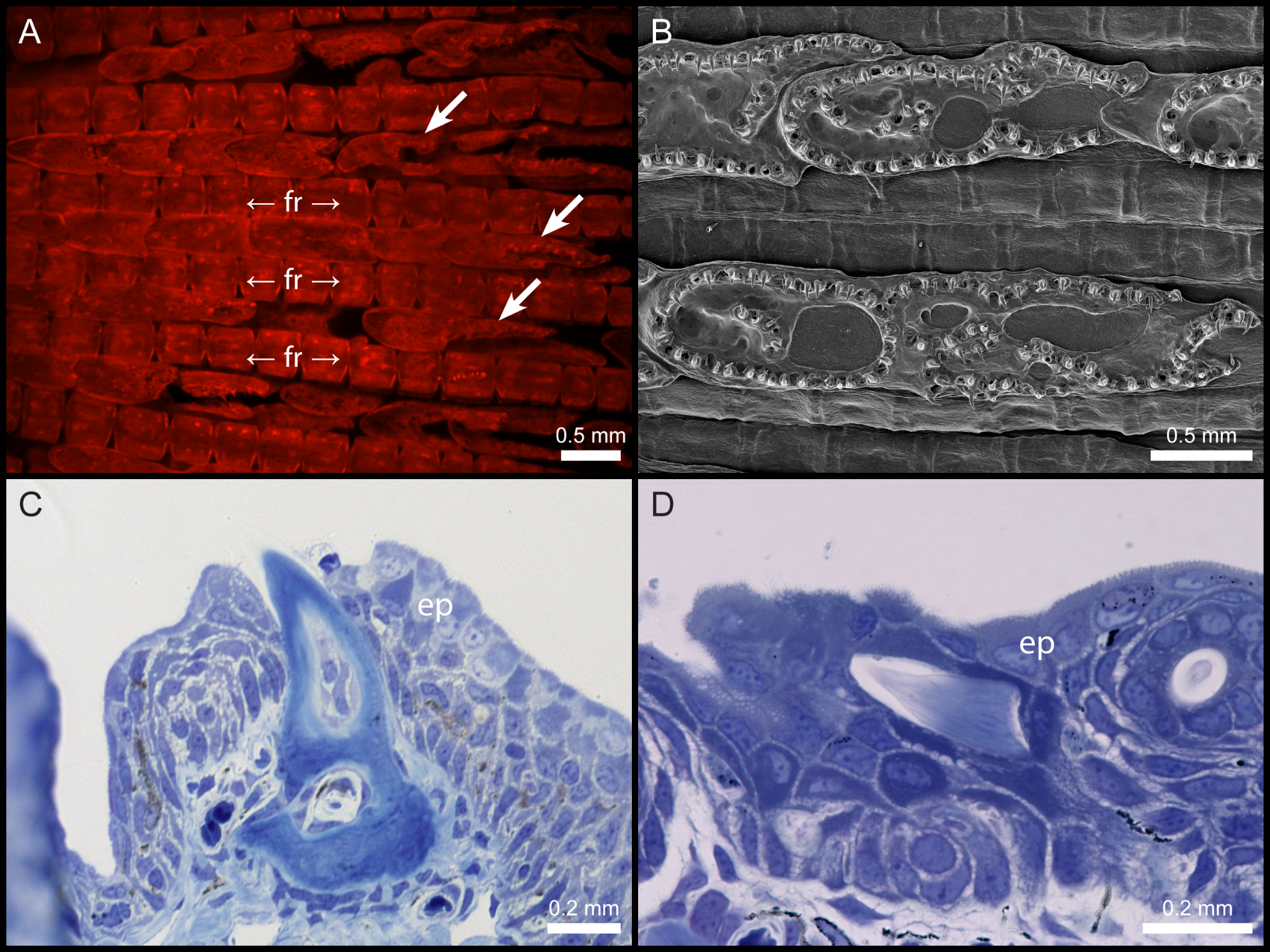


**Suppl. fig. 2** (A) Serial dental plates (arrows) are located also on the dorsal side of the pectoral fin in *P. delhezi*. (B) Merged serial dental plates in the distal region of the pectoral fin in *P. bichir*. (C) Fully formed odontode of dental plate penetrates the epidermis in *P. senegalus*. (D) Presence of the tip of an odontode in the epidermis of *P. senegalus* suggests active resorption rather than shedding. Abbreviations: ep – epidermis; fr – fin ray. Scale bars as indicated.


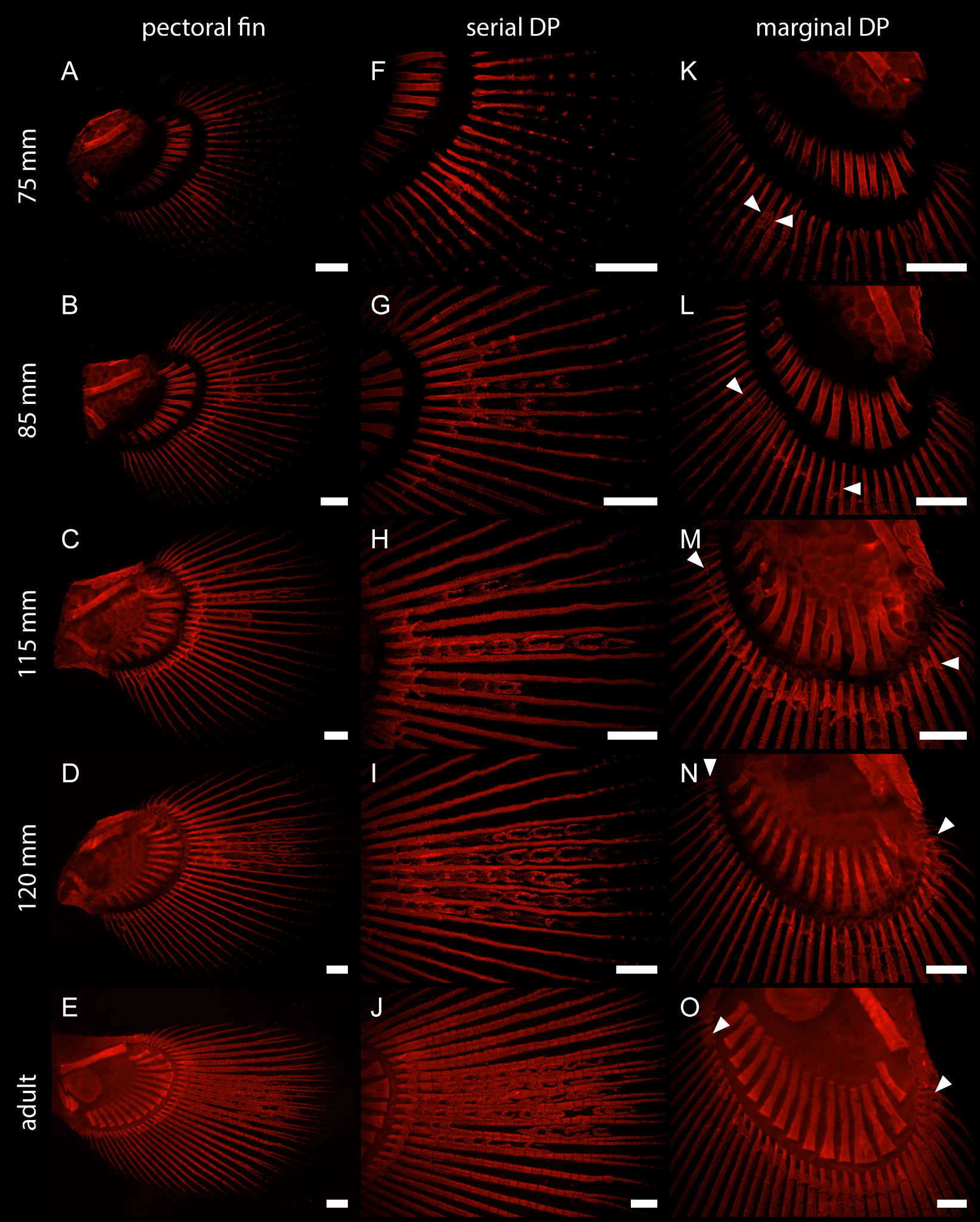


**Suppl. fig. 3** Developmental patterning of dental plates in Senegal bichir. Overall view on pectoral fin (A-E). Development of serial dental plates (F-J) and marginal dental plates (K-O), where the lateral-most extension of the dental plates is indicated with arrowheads. Scale bars = 1 mm.


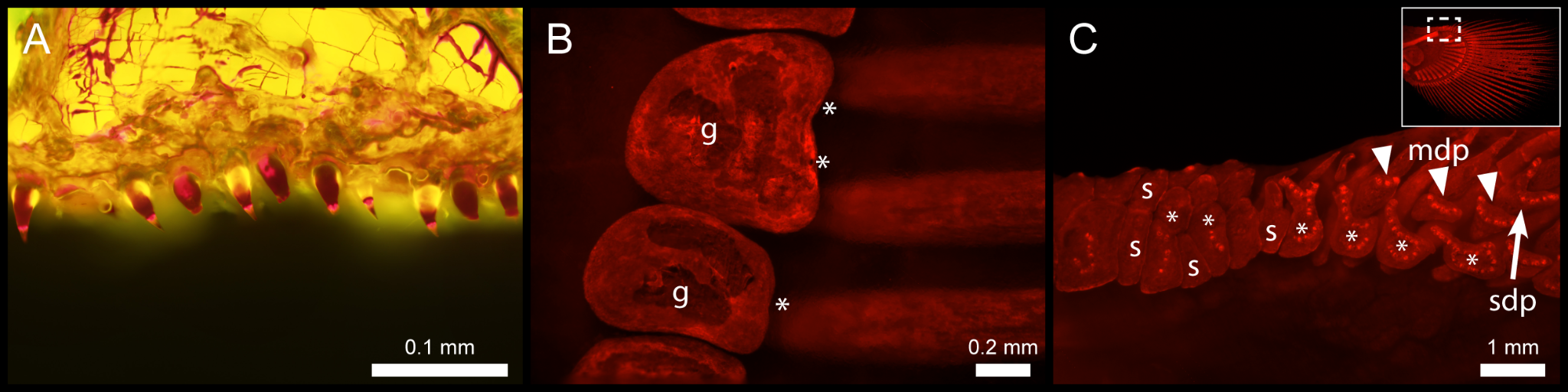


**Suppl. fig. 4** (A) Alizarin stained dental plate shows alternating pattern of odontode replacement. (B) Marginal dental plates of an old bichir possess no odontodes (absence indicated by asterisks). (C) Transitional scales (asterisks) on the ventral side of the pectoral fin (area of the fin indicated in the box). Abbreviations: g– ganoine; mdp – marginal dental plate; s – scale; sdp – serial dental plate. Scale bars as indicated.
